# Integration of Functional Polymers and Biosensors to Enhance Wound Healing

**DOI:** 10.1002/adhm.202401461

**Published:** 2024-09-05

**Authors:** Proma Basu, Aihik Banerjee, Prince David Okoro, Arameh Masoumi, Baishali Kanjilal, Mohsen Akbari, Manuela Martins‐Green, David G. Armstrong, Iman Noshadi

**Affiliations:** ^1^ Department of Bioengineering University of California, Riverside Riverside CA 92521 USA; ^2^ BICURE LLC San Marino CA 91108 USA; ^3^ Department of Mechanical Engineering University of Victoria Victoria BC V8P 5C2 Canada; ^4^ Department of Molecular Cellular and Systems Biology University of California, Riverside Riverside CA 92521 USA; ^5^ Keck School of Medicine of the University of Southern California Los Angeles CA 90033 USA

**Keywords:** animal models, bacteria, biofilm, chronic wounds, DFUs, hydrogels, polymers

## Abstract

Biosensors have led to breakthroughs in the treatment of chronic wounds. Since the discovery of the oxygen electrode by Clarke, biosensors have evolved into the design of smart bandages that dispense drugs to treat wounds in response to physiological factors, such as pH or glucose concentration, which indicate pathogenic tendencies. Aptamer‐based biosensors have helped identify and characterize pathogenic bacteria in wounds that often form antibiotic‐resistant biofilms. Several functional polymers have served as indispensable parts of the fabrication of these biosensors. Beginning with natural polymers such as alginate, chitosan, and silk‐based fibroin, which are biodegradable and absorptive, advances have been made in formulating biocompatible synthetic polymers such as polyurethane and polyethylene glycol designed to reduce non‐specific binding of proteins and cells, making biosensors less painful or cumbersome for patient use. Recently, polycaprolactone has been developed, which offers ductility and a large surface‐area‐to‐volume ratio. There is still room for advances in the fabrication and use of biosensors for wound healing and in this review, the trend in developing biosensors from biomarker detection to smart dressings to the incorporation of machine learning in designing customized wound patches while making application easier is highlighted and can be used for a long time.

## Graphical abstract

The integration of functional polymers and biosensors is advancing wound healing through real‐time monitoring and smart drug delivery. This review highlights recent advancements from natural polymers like alginate and chitosan to synthetic polymers such as polyurethane. It explores innovations in biomarker detection and machine‐learning‐enabled smart dressings poised to significantly enhance chronic wound healing and patient outcomes.

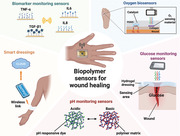

## 1 Introduction

**Figure 1:**
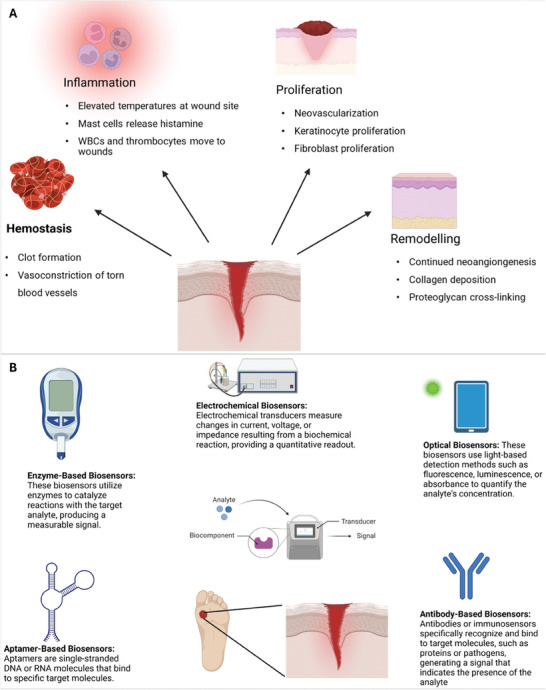
A) Stages of wound healing. Wounds go through four stages of healing, hemostasis, inflammation, proliferation, and remodeling. The key steps at each stage are marked under the stages in the figure. In chronic wounds, however, one or more of these phases are disorganized, occur out of turn or the wound simply never resolves inflammation. The alkaline pH of wounds (pH > 8) is a marker of prolonged unresolved inflammation. Hence, biosensors target pH several times to monitor wound physiology or formulate targets for drug delivery in smart dressings. B) Types of Biosensors in wound healing. An overview of several advances made in detecting wound parameters. Often in biomarker‐based biosensors or ‘smart’ dressings two or more of the technologies are combined to either monitor several parameters simultaneously or couple detection of the parameter with signaling the delivery for drug release into the wound.

Cutaneous wound healing involves completing a series of spatial and temporal processes in order, leading to scar formation and re‐establishment of the epithelial barrier. The first phase of healing is hemostasis. During hemostasis, the formation of a clot consisting of fibrin and platelets stops the bleeding. Platelets release factors that stimulate an inflammatory phase, which results in the chemoattraction of leukocytes to the wound site. Leukocytes are the first to appear. These neutrophils kill pathogens through reactive oxygen species (ROS) and stop wound infection. This is followed by the arrival of monocytes that become proinflammatory macrophages at the wound site, which cleans neutrophil damage through bacterial phagocytosis. Anti‐inflammatory macrophages, which secrete cytokines and growth factors that promote healing, appear in the wound. During the proliferative phase, fibroblasts move into the wound and produce extracellular matrix (ECM) molecules. These provide the required structural and biochemical functions for new wound tissues. During this phase, keratinocytes replicate and migrate to cover the wound, forming the epidermis, whereas the endothelial cells form new microvessels in the wound tissue to bring in nutrients and oxygen. These processes create a “granular appearing” granulation tissue. The final wound‐healing phase is that of remodeling, where surplus cells are killed by apoptosis. The excess ECM‐produced healing tissue formation is removed by phagocytes (**Figure** **1**A). This remodeled wound tissue is the scar tissue.^[^ ^1^ ^]^

This is the pathway for acute wound healing, which leads to impaired healing when disrupted. In such a case, wounds fail to close properly; the wounds do not close properly and there is a failure in the establishment of the skin barrier or the evolution of granulation tissue into scar tissue. Therefore, defective regulation of the complex cellular and molecular processes involved in proper healing can result in wound chronicity.^[^ ^2^, ^3^, ^4^, ^5^, ^6^, ^7^ ^]^ Chronic wounds are characterized by chronic inflammation, a lack of vascularization, and a lack of re‐epithelialization. They are often colonized, and that colonization develops into biofilm‐forming bacteria, resulting in wound chronicity.^[^ ^8^ ^]^

Chronic wounds constitute a global health threat. They impact ≈8.2 million people and cost ≈$28.1–96.8B per year in the US alone.^[^ ^4^ ^]^ A prominent chronic wound type is diabetic foot ulcers (DFUs) are a prominent chronic wound type with a 5‐year mortality rate comparable to that of cancer.^[^ ^9^ ^]^ About 12% of patients with DFUs require amputation, followed by a ≈50% 5‐year survival rate post amputation.^[^ ^4^ ^]^ There is a critical need for a cure for DFUs, given the increase in type II diabetes, which accounts for ≈90–95% of all diabetes types. Diabetes affects 387M people globally and 28M in the US, while pre‐diabetes affects 316M globally and 86M in the US.^[^ ^10^, ^11^ ^]^

There have been many studies to understand the process complexity involved in wound chronicity. Thus far, studies have failed to comprehensively explain the multi‐dimensional process complexity in chronic diabetic wound development or healing. Many treatments, including chemical molecules, growth factors, patches with live cells, ECM patches, and transplanted skin, have been tested without much success.

Biosensors are devices that can detect and measure biological or chemical substances in real time. They often contain a biological component, like an enzyme or antibody, which interacts with the target molecule, generating a signal that can be measured. The concept of biosensors was first proposed in 1906 by Michael Cremer by demonstrating that a difference in the electric potential in a fluid compartmentalized across a glass membrane is directly proportional to the concentration of acid in the liquid.^[^ ^12^, ^13^ ^]^ In 1922, the enzyme invertase was immobilized on aluminum hydroxide and charcoal in bioactive form. Years of formative research culminated in the design of the ‘Clark electrode’ in 1956. Dr. Leland Clarke designed the Clark electrode to deoxygenate his solutions using the enzyme glucose oxidase, which reduces oxygen to hydrogen peroxide as a by‐product. Soon he realized that the electrode could be used alternatively to detect and quantify glucose thus inventing the first biosensor.^[^ ^12^, ^13^ ^]^ The field has seen astronomical growth since then, and technology that converts signals from bio‐analytes to electronic signals has found many applications in biology, such as glucose monitoring biosensors used by diabetic patients, microbe‐based immunosensors, surface plasmon resonance‐based immunosensors, and nano biosensors.^[^ ^14^, ^15^, ^16^ ^]^

## 2 How Do Biosensors Help in Monitoring Wounds?

Based on technology, there are a variety of biosensors such as enzyme‐based, electrochemical, aptamer, and optical biosensors (Figure 1B). Chronic wounds are characterized by high oxidative stress and prolonged inflammation. In the wound environment, specific physiological parameters of the wound provide information crucial to healing and further treatment.^[^ ^17^, ^18^ ^]^ Here we present the technologies mentioned above classified into analytes (as per wound physiology):

### 2.1 pH Sensing

**Figure 2:**
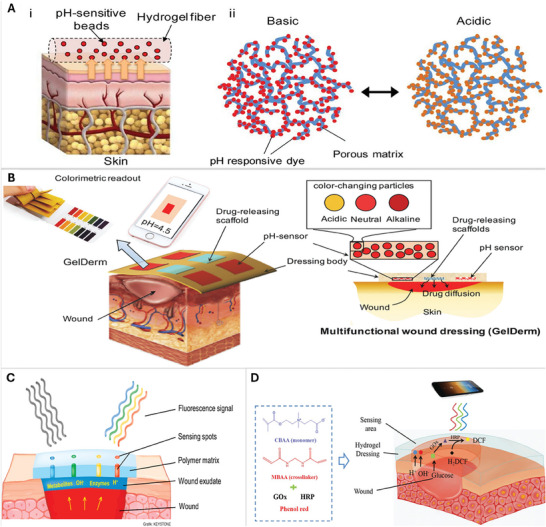
pH monitoring sensors. A) (i) Microfluidic fabrication of hydrogel fibers containing mesoporous silica particles treated with pH‐sensitive dyes. The dye interacts electrostatically with the silica particles and (ii) The color change observed in pH‐responsive beads (silica particles + pH‐responsive dye) when exposed to basic and acidic conditions in the wound. A shift toward acidic pH indicates wound healing. Reproduced with permission.^[^ ^21^ ^]^ Copyright 2016, John Wiley and Sons. B) GelDerm is 3D bioprinted and consists of ion exchange beads doped with pH‐responsive dyes that were loaded onto the four corners of the patch with two drug‐eluting scaffolds in the center dispensing a pre‐determined concentration of gentamicin to treat pathogenic bacteria in the wound. Reproduced with permission.^[^ ^22^ ^]^ Copyright 2017, John Wiley and Sons. Glucose monitoring sensors. C) Glucose sensing enzymes, glucose oxidase, and horseradish peroxidase are immobilized on an alginate matrix along with a pH‐sensitive fluorescent dye, carboxynaphthofluorescein. Together the functionalized matrix formed an optical biosensor. Reproduced with permission.^[^ ^24^ ^]^ Copyright 2017, Elsevier. D) A zwitterionic poly‐carboxybetaine (PCB) hydrogel encapsulates phenol red and two enzymes, horseradish peroxidase and glucose oxidase, to determine pH and glucose concentration simultaneously. The readings can be recorded with a smartphone and analyzed in MATLAB. Reproduced with permission.^[^ ^25^ ^]^ Copyright 2019, John Wiley and Sons.

pH is an important parameter in wound healing dynamics. Chronic wounds are more alkaline (pH 7–9) than acute healing wounds.^[^ ^17^, ^18^, ^19^, ^20^ ^]^ Biosensing patches can measure and signal the pH of the wound. For example,^[^ ^21^ ^]^ designed a microfluidic system for hydrogel‐based fibers that could be assembled into a wearable wound patch (**Figure** **2**A). A hydrogel made with a mix of alginate and glycerol was linked to mesoporous silica particles with pH‐responsive dyes. The best response time was exhibited by 800 µm diameter fibers when tested in a range of pH 6.5 to 9. In vitro testing of hydrogel including pH‐responsive beads in human‐derived keratinocytes showed no noticeable difference in viability compared to control except when pH‐sensitive dye residues leaked from the fibers. The reaction produced fluorescence which can be measured and detected by smart phones hence circumventing the need and time for expensive imaging techniques.^[^ ^21^, ^22^ ^]^ designed a wound patch that simultaneously detects the pH and releases a drug at the wound site for redressal in case of abnormal pH detection. The patch called ‘GelDerm’ also utilizes an alginate, glycerol composite for the hydrogel but instead of a microfluidic device, it is fabricated with a 3D bioprinter. Ion exchange beads doped with pH‐responsive dyes were loaded onto the four corners of the patch with two drug‐eluting scaffolds in the center, dispensing a predetermined concentration of gentamicin to treat *Pseudomonas aeruginosa* (*P. aeruginosa)* and *Staphylococcus aureus* (*S. aureus)* infection in the wound (Figure 2B). The patch successfully identified a range of pH 4–9. Biocompatibility of the patches was assessed using keratinocytes and skin fibroblasts. Both showed no difference in viability compared to the control as there was no leakage of dye from GelDerm.^[^ ^22^ ^]^ The color change is again documented and quantified by a smartphone, as the patch is also transparent. Biosensors have evolved to detect more than one physiological factor at a time (pH, glucose, oxygen, etc.), which also improves treatment.

### 2.2 Glucose Monitoring

Around 415 million patients suffer from diabetes worldwide, of them 15–20% develop chronic wounds which can lead to limb amputations. To treat diabetic foot ulcers (DFUs) in humans, continuous glucose monitoring (CGM) systems, which were originally developed for diabetes management, were adapted for wound applications. These biosensors enable real‐time monitoring of glucose levels in wounds as they directly influence bacterial growth and reflect the physiological conditions of the patient. As pH and glucose are often the parameters in the wound that fluctuate the most, in recent years biosensors have combined the detection of pH and glucose.^[^ ^23^ ^]^ designed a sensor consisting of a polyphenyl boronic acid covalently bonded to porous silicon films (pSi‐PVPBA) which change in thickness in response to changing pH and glucose concentration in the wound. Polymer thickness was measured by interferometric reflectance spectroscopy of the composite film. The effective optical thickness was tested on chronic wound fluid collected from patients. The readily available commercial glucose monitor can only measure glucose in the range of s 0.6–33.3 mm and is not suitable for measurement in wound fluid as it is optimized for blood. The change in effective optical thickness of the pSi‐PVPBA was tested on the wound fluid and in wound fluid spiked with 10 mm glucose and the change in thickness was significantly different between non‐spiked and spiked wound fluid.^[^ ^23^, ^24^ ^]^ immobilized glucose‐sensing enzymes, glucose oxidase, and horseradish peroxidase, on an alginate matrix. A pH‐sensitive fluorescent dye, carboxynaphthofluorescein, was coupled to it forming an optical biosensor to monitor wound healing (Figure 2C). The enzymes were stable on immobilization. The authors used biocompatible sodium alginate as the hydrogel and tested its effectiveness on artificial wound fluid. The pH detection system was also signal reversible when subjected to changing pH.^[^ ^24^, ^25^ ^]^ formulated a zwitterionic hydrogel to encapsulate phenol red, to determine pH, and two enzymes, horseradish peroxidase and glucose oxidase, to determine glucose concentration simultaneously in the wound. Changes in pH and glucose levels are monitored by pictures taken with a smartphone and analyzed in MATLAB (Figure 2D). Authors showed that both horseradish peroxidase and glucose oxidase showed enhanced activity and stability in the presence of zwitterionic poly‐carboxybetaine (PCB) hydrogel which is also known to be biocompatible.^[^ ^25^ ^]^ In a more recent study,^[^ ^26^ ^]^ have expanded upon the functionality of their innovative wound dressing, ‘GelDerm’, through the integration of glucose sensors alongside antibiotics/growth factor‐releasing components. This enhancement reportedly not only broadened GelDerm's capacity for microbial screening but also enhanced its therapeutic efficacy. The incorporation of an array of glucose sensors into the dressing was shown to facilitate real‐time monitoring of glucose concentration at the wound site. These sensors were shown to be functional across various temperature ranges and storage durations. Furthermore, the integration of growth‐factor‐releasing components within GelDerm serves to expedite the wound‐healing process. The resulting GelDerm was reported to exhibit dual functionality, acting both as a physical barrier against pathogens and as a catalyst for wound healing, irrespective of the wound's infectious status.^[^ ^26^ ^]^

### 2.3 Oxygen Sensing

**Figure 3:**
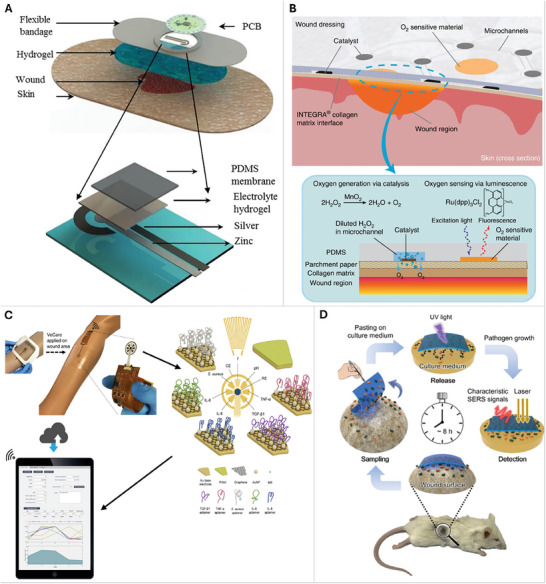
Oxygen sensing biosensors. A) The smart dressing utilizes an electrochemical galvanized cell built on parylene‐C. Potassium hydroxide functions as the electrolyte, silver, and zinc were used as electrodes, and PDMS was used as the polymer in the dressing. Oxygen levels in the wound were uploaded by wireless telemetry or Bluetooth making the setup a wearable smart bandage. Reproduced with permission.^[^ ^27^ ^]^ Copyright 2015, IEEE. B) Fabricated using a microfluidic network, this smart bandage is made of parchment paper coupled to a dermal regeneration matrix made with collagen and glycosaminoglycan that conforms flexibility to the bandage. Potassium permanganate catalyst is used on 3% hydrogen peroxide for oxygen delivery. Reproduced.^[^ ^28^ ^]^ Biomarker sensing biosensors. C) VeCare combines the detection of inflammatory mediators like tumor necrosis factor α (TNF‐α), interleukin‐6, interleukin‐8, growth factor TGF‐β, detection of *S. aureus*, and temperature and pressure in venous ulcers. The wound fluid is collected by a microfluidic system and tested using aptamers designed for each of the biomarkers. Reproduced.^[^ ^30^ ^]^ D) The ‘Three‐in‐one’ adhesive tape to sample the presence of *P. aeruginosa* and *S. aureus*. The process from sampling to confirmation using Raman spectroscopy after allowing the culture to grow is 8 h which is significantly shorter than PCR or colony culture. Reproduced with permission.^[^ ^33^ ^]^ Copyright 2019, American Chemical Society.

Oxygen plays a crucial role in wound healing, and biosensors capable of measuring oxygen levels in wounds become an important tool.^[^ ^27^ ^]^ reported an electrochemical galvanized cell built on parylene‐C that functions as an oxygen sensor for wounds. Potassium hydroxide functions as the electrolyte and silver and zinc were used as electrodes. The dressing was attached to a poly(dimethylsiloxane) membrane (**Figure** **3**A). Oxygen levels in the wound were uploaded using wireless telemetry or Bluetooth, making the setup a wearable smart bandage. The performance of the smart bandage in measuring oxygen showed a linear relationship between the oxygen concentration and the current generated. However, the smart bandage was tested in a simulated wound environment only.^[^ ^27^, ^28^ ^]^ integrated oxygen sensing and delivery to a wound using a parchment paper substrate with an embedded microfluidic network. The parchment paper patch is made flexible by coupling it to a dermal regeneration matrix made with collagen and glycosaminoglycan. Oxygen sensing was performed by phosphorescence using a ruthenium compound. Potassium permanganate catalyst is used on 3% hydrogen peroxide for oxygen delivery (Figure 3B). The patches were tested both in vitro and in vivo. L‐929 mouse fibroblast cells were used for in vitro testing and showed that patches should be washed in Hank's buffered saline solution followed by a complete growth medium to reduce cytotoxicity. Post‐washing cell attachment was calculated at > 85% showing minimal cytotoxicity. Eight‐week‐old male SKH1 (hairless) mouse was used to test a miniature patch on a splinted excisional wound healing model. The wound healing rate in wounds with the oxygenating patch was found to be slower compared to the non‐oxygenating patch but both wounds were closing by 14 days post‐surgery.^[^ ^28^, ^29^ ^]^ used boron nanoparticles that exhibit oxygen‐independent fluorescence and oxygen‐dependent phosphorescence. The ratio between the fluorescence and phosphorescence is then used as a relative measure of oxygenation in the wound. For in vivo testing, 3–4 month‐old female C57Bl/6 mice with full‐thickness dermal wounds and one female Yorkshire swine with full‐thickness excisional wound were used. Acute wounds in mice treated with the patch showed a decrease in wound area with an increase in fluorescence to phosphorescence ratio over 3 days post‐wounding. As only one swine was used for the porcine wound model, no conclusions on wound closure were made but the authors did report an increase in oxygen partial pressure in the wound with increasing fluorescence to phosphorescence ratio.^[^ ^29^ ^]^

### 2.4 Biomarker Detection

In recent years, biosensors have been employed to detect specific biomarkers indicative of wound healing status. Cytokines, growth factors, and proteases associated with inflammation, lactic and uric acid, and bacterial metabolites can be measured using biosensor platforms. This enables the assessment of wound progression and the identification of potential complications.^[^ ^30^ ^]^ made a dressing (VeCare) that combines the detection of inflammatory mediators such as tumor necrosis factor α (TNF‐α), interleukin‐6, interleukin‐8, growth factor TGF‐β, detection of *S.aureus*, and temperature and pressure in venous ulcers. The wound fluid is collected by a microfluidic system and tested using aptamers designed for each of the biomarkers (Figure 3C). Graphene and gold nanoparticle composite were used as electrodes for electrochemical analysis of the aptamers exposed to wound fluids.^[^ ^30^ ^]^ The authors further reported that the aptasensors showed good long‐term stability over 4 weeks with drifts less than 5%. Components of an open‐source universal wireless electrochemical detector developed by^[^ ^31^ ^]^ were adopted in the design to include a potentiostat for pH sensing and a microcontroller for wireless electrochemical detection of the different wound parameters.^[^ ^31^ ^]^ Temperature sensing was incorporated in the design using a Wheatstone bridge differential amplifier configuration by measuring resistance. The effectiveness of the biomarker analytical dressing was tested in full‐thickness excisional wounds on 10‐12‐week‐old male Institute of Cancer Research outbred mice. The patch showed proper monitoring of pH, temperature, mouse TNF‐α, and *S. aureus*, including a reduction in pH corresponding to re‐epithelialization in 5 days of monitoring post‐wounding. All data was collected and reported via custom‐designed MATLAB applications.^[^ ^30^, ^32^ ^]^ designed wearable carbon ultramicroelectrode arrays (CUAs) on flexible multi‐component substrates made from polymethylmethacrylate dissolved in chlorobenzene. Three biomarkers pyocyanin, a metabolite produced by common wound pathogen *P. aeruginosa*, uric acid, and nitric oxide are detected using this biosensor. Data on the biomarkers was collected using square wave voltammetry, in which the CUAs participate as an electrode, as well as UV‐Vis measurements for pyocyanin and nitric oxide only. The patch was tested on wound fluid simulant containing RAW 264.7 macrophage cells and showed increasing current in response to an increase in the concentration of pyocyanin, uric acid, and nitric oxide in square wave voltammetry data.^[^ ^32^ ^]^ Bacterial load, particularly pathogenic bacteria like *Escherichia coli (E. coli)*, *S. aureus*, and *P. aeruginosa* can be detected by biosensors as well.^[^ ^33^ ^]^ designed a “Three‐in‐one” adhesive tape to detect the concurrent presence of *S.aureus* and *P.aeruginosa* in the wound. The adhesive tape samples the bacteria on the wound and is then subsequently processed by Raman characterization of the bacteria sampled on gold nanostars held between two pieces of graphene. The analytical capabilities of the “Three‐In‐One” adhesive tape were tested on a burn wound model in mice. Seven‐week‐old male BALB/c mice were used for creating skin burn wounds and the wound was spiked with 1 × 10^6^ cfu mL^−1^ of *P. aeruginosa* and *S. aureus*. The readings to quantify *P. aeruginosa* and *S. aureus* were found to be consistent with other applications of Raman spectroscopy in identifying bacterial count, also, this method was found to be simplified and time‐effective compared to traditional methods like colony culture and PCR (Figure 3D).^[^ ^33^ ^]^ These pathogenic bacteria often give rise to biofilm formation hence early detection to inform the course of treatment is invaluable in treating chronic wounds.

Increasingly, biomarker detection is being used in the formulation of smart dressings which often couple the detection with machine learning algorithms or timed release of drugs for continuous redressal of the wound and will be discussed in the next section.

### 2.5 Smart Dressings

**Figure 4:**
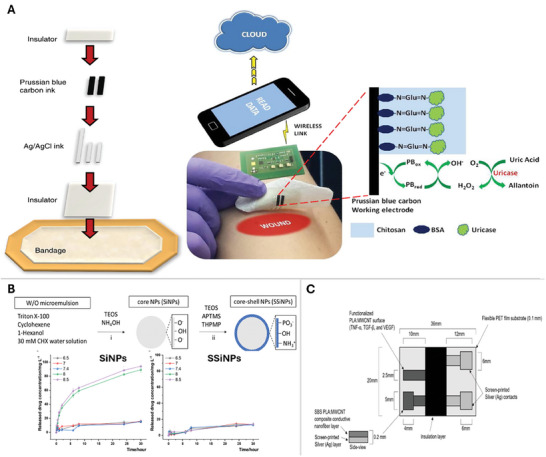
Smart dressings in wound biosensing. A) 3D printed smart bandage uses an immobilized uricase enzyme to determine uric acid concentration in the wound. The Prussian blue working electrode catalyzes the oxidation of uric acid to hydrogen peroxide thereby generating a reduction current proportional to the amount of uric acid in the wound. Reproduced with permission.^[^ ^34^ ^]^ Copyright 2015, Elsevier.^[^ ^34^ ^]^ B) Shows the fabrication of chlorhexidine dispensing silica nanoparticles. The antibiotic is only dispensed at alkaline pH conditions native to chronic wounds and the line graphs show that a single coating of microemulsion on core silica nanoparticles is more efficient in drug release compared to a double coating. Reproduced.^[^ ^35^ ^]^ C) The biomarker sensor was made of a nanofiber composite mounted on a polyethylene surface. The nanofiber composite was loaded with antibodies to quantitatively measure the amount of TNF‐α, TGF‐β, and VEGF via an electrochemical response. The sensor is novel in its use of machine learning from light and thermal photographs of venous ulcers to determine the stage of wound progression. Reproduced.^[^ ^18^ ^]^

Advances in biosensor technologies have led to the integration of sensors into wound dressings as in “smart dressings” that monitor and signal changes in the wound for timely infection redressal. The parameters monitored entail temperature, moisture, pH, bacterial presence, and other markers like uric acid concentration, especially in chronic wounds.^[^ ^23^, ^34^ ^]^ devised a smart screen‐printed bandage with an immobilized *Candida*‐derived uricase enzyme coupled with a catalytic Prussian blue transducer. The functional electrode was made by drop‐casting a solution of BSA, glutaraldehyde, and uricase in PBS. The electrode was coated with a biocompatible chitosan polymeric layer to reduce the leaching of sensor components into the wound. Uricase catalyzes the oxidation of uric acid to form hydrogen peroxide which in turn is reduced by Prussian Blue, thereby generating a reduction current proportional to the amount of uric acid in the wound (**Figure** **4**A). This current was read by a wearable potentiostat as well as a CHI 440 electrochemical analyzer. The concentration was measured reproducibly by both instruments. However, the study did not report any biocompatibility studies using cell culture or animal models.^[^ ^34^ ^]^ Such chronoamperometric biosensors can monitor and transmit reports of chronic wounds facilitating timely intervention.^[^ ^35^ ^]^ designed chlorhexidine‐loaded silica nanoparticles embedded in alginate polymer hydrogel that are designed to deliver the antibacterial chlorhexidine when exposed to alkaline wound pHs (>8) (Figure 4B). The silica nanoparticles released chlorhexidine, verified with Fourier transform infrared analysis which underscored significant antibacterial properties toward *E.coli* (gram‐negative) and *S.aureus* (gram‐positive) in culture. These two bacteria are also two of the most abundant in wounds. Cytotoxicity of the nanoparticles was tested using normal Human dermal fibroblasts and compared to the positive control chlorhexidine‐loaded nanoparticles showed cell viability similar to the negative control (70% cell viability).^[^ ^18^, ^35^ ^]^ published a proof‐of‐concept protocol on a machine learning algorithm‐based sensing protocol, based on imagery and wound biomarkers to classify wound progression. The biomarker sensor was made of a nanofiber composite mounted on a polyethylene surface. The nanofiber composite was loaded with antibodies to quantitatively measure the amount of TNF‐α, TGF‐β, and VEGF via an electrochemical response normalized to the impedance of the wound bed (Figure 4C). The design was tested on patients with chronic venous leg ulcers. The wound was visually assessed and scored using the Bates Jensen Wound Assessment Tool (BWAT) scale. The design implements machine learning in wound care at the patient level and requires fewer images of the wound as a pre‐trained model gathers information on wound color, and margins using the convolutional neural networks only for specific wound characteristics that vary from patient to patient.^[^ ^36^ ^]^

The aforementioned biosensors in terms of technology are itemized in **Table** **1**. Going forward artificial intelligence models utilizing machine learning to adapt and customize treatment to individual patients with wounds are the future. Data mining techniques from each type of chronic wound can be gathered from the published biosensor data in various in vitro and in vivo models. Feature extraction using principal component analysis (PCA), or discrete Fourier transform for time‐course data can be used. These features can be used to predict wound progression using traditional regression techniques. However even if properly labeled data is not available, databases can be scanned for pattern discovery using hierarchical clustering or DBSCAN or Gaussian mixture model to inform wound management (http://hdl.handle.net/10400.14/34902, diss).^[^ ^37^, ^38^, ^39^ ^]^

**Table 1:** Classification of biosensors customized for addressing challenges in wound healing.

Type of sensor	Analyte	Biocomponent / Sensor	References
Aptamer	TNF‐α, IL6, IL8, TGF‐β, *S.aureus*	Aptamers	[30]
Enzyme	glucose	glucose oxidase, horseradish peroxidase	[24]
	glucose	glucose oxidase, horseradish peroxidase	[25]
Electrochemical	oxygen	parylene‐C	[27]
	pH	potentiostat	[30, 31]
	temperature	Wheatstone bridge differential amplifier	[30, 31]
	uric acid	uricase on a polymer matrix	[19]
	uric acid	uricase enzyme coupled with a catalytic Prussian blue transducer	[34]
	pyocyanin, uric acid, nitric oxide	carbon ultramicroelectrode arrays	[32]
Optical	pH, glucose	polyphenylboronic acid covalently bonded to porous silicon films	[23]
	pH	carboxynaphthofluorescein	[24]
	pH	phenol red	[25]
	pH	fluorescein isothiocyanate	[35]
	pH	brilliant yellow dye encapsulated in anion exchange resin beads	[21]
	pH	GelDerm colorimetric measurement	[22]
	oxygen	ruthenium compounds	[28]
	oxygen	boron nanoparticles	[29]
	*S.aureus* and *P.aeruginosa*	gold nanostars held between two pieces of graphene	[33]
Antibody	TNF‐α, TGF‐β and VEGF	nanofiber composite loaded with antibodies	[18]

## 3 Polymers in Wound Biosensing and Management

Wound care and tissue engineering have witnessed significant advancements with the integration of natural and synthetic polymers in wound biosensing and management.^[^ ^40^ ^]^ This review focuses on the utilization of various natural biopolymers, including alginate, gelatin, silk fibroin, chitosan, and cellulose, in wound biosensing applications. Additionally, synthetic polymers, such as polyurethane, polyvinyl alcohol, polyethylene glycol, and polycaprolactone, are discussed for their role in wound biosensing modalities. The remarkable properties of these polymers, including biocompatibility, biodegradability, and tunable material properties, make them promising candidates for wound management and biosensing applications (**Table** **2**). Several examples of polymer‐based biosensors are presented to highlight their efficacy in wound monitoring, infection detection, and wound healing.

**Table 2:** Functional polymers in biosensor fabrication.

Polymers in wound dressing	Advantages
**Natural polymers**	
Alginate	cost‐effective, forms ionotropic hydrogels
Gelatin	RGD peptides, tissue adhesiveness, thermo‐sensitivity
Silk Fibroin	bioactive, widely available, absorptive, cost‐effective
Chitosan	non‐toxic, biodegradable, electronic conductivity
Cellulose	biodegradable, biocompatible, low cytotoxicity
**Synthetic polymers**	
Polyurethane	biocompatible, biodegradable
polyvinyl alcohol	non‐toxic, biocompatible, elastic, water absorption
Polyethylene Glycol	hydrophilic, biocompatible, inert in aqueous media, reduces nonspecific binding of proteins and cells
Polycaprolactone	biodegradable, biocompatible, high plasticity, ductility, large surface‐area‐to‐volume ratio

Natural polymers offer several benefits such as ease of availability, high bioactivity, biocompatibility, biodegradability, and superior cytocompatibility, and are usually closely mimetic of native extracellular matrix (ECM). However, natural polymers can suffer from limitations such as high cost, limited tunability of physical, chemical, and mechanical properties, and can elicit an immune response due to their protein content. Additionally, natural polymer‐based solutions can suffer from significant batch‐to‐batch variability owing to their complex chemistry. On the other hand, synthetic polymers, owing to their defined chemical structures, allow for a high degree of physical, chemical, mechanical, and surface modifications to tailor them to specific applications, besides being low‐cost, biocompatible, and less prone to systemic degradation or immunogenic reactions. Specifically, synthetic polymers tend to be biologically inert which can hinder their biological applications.^[^ ^41^, ^42^ ^]^ As such, the choice of materials for biological applications, such as wound care and biosensing, is dictated by the nature of the application, with no one‐size‐fits‐all solution yet. Oftentimes, the mixing of natural and synthetic polymers is done in a controlled fashion to achieve composite materials, combining the advantageous properties of their natural and synthetic components. Since this review focuses on polymeric materials that are attractive for wound biosensing applications, the following section highlights the fabrication of wound biosensors using natural and synthetic polymers. Moreover, studies showcasing wound biosensors based on a composite between natural and synthetic polymers are also discussed.

### 3.1 Natural Polymers

#### 3.1.1 Alginate‐Based Wound Biosensors

Alginate, a cost‐effective biopolymer derived from brown seaweed and specific bacteria, has been extensively studied for its unique structure and properties.^[^ ^43^, ^44^, ^45^ ^]^  Briefly, alginate is readily available, non‐toxic, highly biocompatible, easily tunable, and exhibits high absorption, swelling, and antibacterial activities. These properties make alginate highly conducive to wound healing applications. Particularly, its ability to form ionotropic hydrogels when crosslinked by divalent or trivalent cations makes it an excellent base material for biosensing applications.^[^ ^46^, ^47^ ^]^

**Figure 5:**
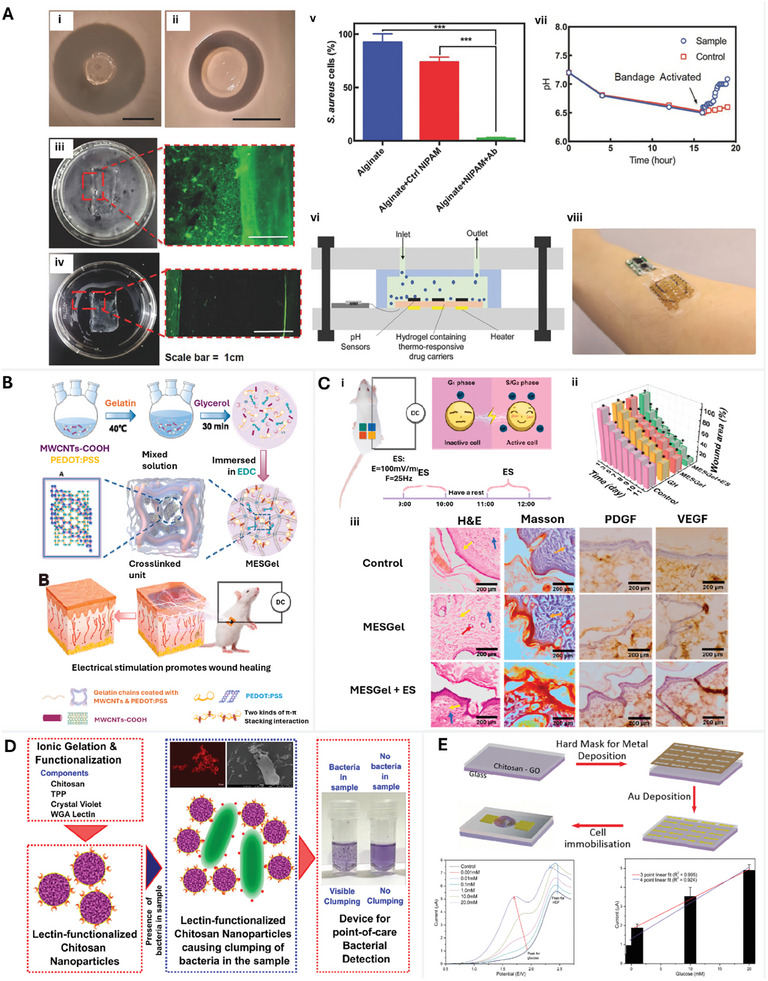
Natural polymer‐based systems for wound biosensing and antibacterial activity. Alginate‐based polymer systems. A) Assessment of antibacterial properties in wound management. i, ii) Diffusion assay comparing antibacterial‐releasing hydrogel and antibiotic‐free hydrogel control. iii, iv) Biofilm visualization on control and antibacterial patches, with live bacteria denoted in green. v) CFU assay for *S. aureus* interactions with cefazolin. vi) In vitro setup for *S. aureus* culture with real‐time pH monitoring and subsequent heater activation. vii, viii) Integration of all components and patch placement on the author's hand. Reproduced with permission.^[^ ^50^ ^]^ Copyright 2018, John Wiley and Sons. Gelatin‐based bio‐electronic hydrogels. B) Overview of MESGel synthesis and its role in wound healing acceleration via electrical stimulation. Reproduced with permission.^[^ ^56^ ^]^ Copyright 2021, Elsevier. C) In vivo wound healing assessment in SPF rats subjected to full‐thickness skin injuries: i) Experimental design for skin wound intervention. ii) Quantitative representation of wound size reduction over 10 days across treatments. iii) Histological and immunohistochemical examination on day 20: Fibroblasts (yellow arrow), blood vessels (red arrow), neutrophils (blue arrow), collagen presence (orange arrow), and markers of PDGF and VEGF (both shown as brown‐stained regions). Reproduced with permission.^[^ ^56^ ^]^ Copyright 2021, Elsevier. Chitosan‐based sensing platforms for bacterial detection and diabetic wound monitoring. D) Illustration of the lectin‐functionalized chitosan nanoparticle design with crystal violet integration. Their agglutination behavior signals bacterial infections through visible clumping distinct from nonbacterial samples. Reproduced with permission.^[^ ^67^ ^]^ Copyright 2022, American Chemical Society. E) Construction overview of the ultra‐slim CS‐GO sensor embedded with gold microgap (60 µm) electrodes. Linear sweep voltammetry (LSV) showcases the responsiveness of the human dermal fibroblast (HDF)‐immobilized CS‐GO substrate to varied glucose concentrations, providing accuracy over a broad spectrum. Data presented as mean ± standard deviation for three replicates. Reproduced.^[^ ^68^ ^]^

Biosensors based on ionotropic alginate hydrogels allow the target analyte to bind within the hydrogel, leading to the generation of measurable signals using enzymes, nanoparticles, or responsive polymers. Notably, these biosensors have demonstrated outstanding performance in detecting glucose and lactate in real samples like human sweat and blood, showcasing their excellent sensitivity.^[^ ^48^, ^49^ ^]^ Moreover, significant strides in this domain have yielded a sophisticated smart bandage tailored for chronic wound monitoring as demonstrated by.^[^ ^50^ ^]^ The reported ultra‐thin (<3 mm) bandage integrated pH and temperature sensors, a microheater with a 20‐ohm resistance, thermo‐responsive poly(N‐isopropyl acrylamide) (PNIPAM)‐based drug carriers embedded in an alginate hydrogel patch, and an electronics patch that orchestrates drug release through sensor‐driven thermal actuation. This innovation enabled real‐time wound monitoring, in situ infection identification, and on‐demand drug release, holding substantial promise for healthcare applications. The potentiometric pH sensor, fabricated on a flexible parylene substrate, demonstrated a linear response and sensitivity of −50 mV pH^−1^. Electrodes comprising carbon/polyaniline (PANI) and silver/silver chloride were utilized as the working and reference electrodes, respectively, with PANI as a positive exchange membrane. The pH sensor's input triggered the hydrogel patch to release antibacterial drugs (**Figure** **5**A). The wireless regulation of sensor data and drug delivery was executed via a Bluetooth module integrated into the electronics patch. The bandage's effectiveness and safety were substantiated through in vitro bacterial assays, antibiotic elution assessments, a conventional scratch wound healing analysis, and cytocompatibility investigations involving human keratinocytes. Beyond pH, temperature sensors, and antibiotics, the platform was proposed to have the capacity for additional sensors, drugs, and growth factors designed to address specific healing markers and targeted treatments.^[^ ^50^ ^]^ However, the application of this intelligent wound bandage to a chronic wound animal model in an in vivo setting remains an unexplored frontier.

#### 3.1.2 Gelatin‐Based Wound Biosensors

Gelatin, denatured collagen, is widely used in tissue engineering and wound healing due to its RGD peptides, tissue adhesiveness, and thermo‐sensitivity.^[^ ^51^, ^52^ ^]^ Its excellent biocompatibility makes it an ideal choice for biosensors in various biological applications. Gelatin exhibits excellent hemostatic, anti‐microbial, and anti‐inflammatory properties, and promotes cellular migration, proliferation, vascularization, and epithelialization, making it very attractive for wound repair and regeneration.

Gelatin‐based biosensors have been successfully used in detecting analytes such as glucose, proteases, and hydrogen peroxide for the last four decades.^[^ ^53^, ^54^, ^55^ ^]^ Recent studies have showcased the use of gelatin‐based biosensors in wound status monitoring. As exemplified by,^[^ ^56^ ^]^ an integrative crosslinking methodology employing N‐(3‐Dimethylaminopropyl)‐N′‐ethylcarbodiimide hydrochloride (EDC) was harnessed to craft a bioinspired 3D‐scaffold based on gelatin. This scaffold integrated water‐dispersible conducting polymer complex poly(3,4‐ethylenedioxythiophene)‐poly(styrenesulfonate) (PEDOT:PSS) and carboxyl‐functionalized multi‐walled carbon nanotubes (MWCNTs‐COOH). Dubbed ‘MESGel,’ this gelatin‐based structure was engineered to embrace mechanical flexibility, electroactivity, and the distinctive property of mechanical self‐healing. The synthesis of MESGel was achieved via a streamlined ‘one‐pot’ methodology, integrating physical doping and chemical crosslinking in a synergistic manner. This concerted approach facilitated a diverse spectrum of molecular interactions within MESGel, leading to the establishment of a complex 3D network within the bio‐inspired hydrogel framework (Figure 5B). Within the domain of wound management, MESGel has introduced a revolutionary paradigm by amalgamating multifunctional motion sensing with the acceleration of skin wound healing through electrical stimulation, as demonstrated by its reparative effects in a rat model featuring full‐thickness skin defects (Figure 5C). MESGel has exhibited not only a significant reduction in wound area but also enhanced granulation tissue formation, augmented collagen deposition, stimulated vascularization, and expedited re‐epithelialization. The harmonious integration of electroactivity and bioelectronics within MESGel has been further corroborated by its ability to promote in vitro proliferation of Chinese hamster lung (CHL) cells.^[^ ^56^ ^]^ However, the performance of MESGel in chronic wound animal models remains unexplored, presenting a potential avenue for future investigation.

In another notable study by,^[^ ^57^ ^]^ a colorimetric pH sensor was conceived through a dynamic strategy involving the active ingredient‐based polyelectrolyte complex methodology. Biopolymers, specifically the polyampholytic gelatin and the anionic polysaccharide sodium alginate, were harnessed to construct a high‐molecular‐weight hydrogel bead. Capitalizing on gallic acid, an adaptable natural antioxidant, as a discernible indicator for detecting wound infections, the hydrogel bead showcased an array of extraordinary attributes. Its inherent pale yellow/off‐white tint in acidic conditions remained unaltered, yet it underwent a rapid transformation to a distinct hue in basic pH environments (above 7.4), serving as an immediate bacterial infection indicator. This color transition manifested instantly. Beyond its visual utility, the hydrogel bead exhibited impressive antioxidant potential, established non‐cytotoxicity, and adeptly absorbed significant quantities of wound exudates.^[^ ^57^ ^]^ It is worth noting, however, that the sensor's performance in an in vivo setting, specifically within a chronic wound animal model, has yet to be investigated.

#### 3.1.3 Silk Fibroin‐Based Wound Biosensors

Silk fibroin (SF), derived from silkworms and other insects, is obtained through a degumming process, removing the gum‐like structure sericin.^[^ ^58^, ^59^ ^]^ SF's remarkable properties have made it suitable for various wound treatment approaches, including therapeutic delivery systems.^[^ ^60^ ^]^ Its natural bioactivity, biocompatibility, wide availability, and cost‐effectiveness make it an appealing candidate for wound healing and tissue engineering. Specifically, SF can induce wound healing by increasing cell growth, migration, and proliferation of various cells involved in different stages of the wound healing process, leading to optimal tissue regeneration. SF has also proven to be a versatile and reliable material for enzyme immobilization in biosensors, enabling efficient enzyme absorption and high sensitivity in various applications.

SF‐based biosensors have shown success in systemic monitoring, wound healing, and continuous temperature monitoring, highlighting their practicality and potential.^[^ ^61^, ^62^, ^63^, ^64^ ^]^ Notably, a colorimetric SF biosensor was developed to detect reactive oxygen species (ROS) in wounds, offering visible indications for timely and targeted treatments.^[^ ^63^ ^]^ In the study,^[^ ^63^ ^]^ harnessed the electrospinning technique to fabricate nanofibrous silk fibroin mats. These mats were loaded with Amplex red dye, facilitating the real‐time assessment of oxidative stress within wounds via the conversion of Amplex to resorufin in the presence of hydrogen peroxide (H_2_O_2_). In vitro color sensing experiments showcased that H_2_O_2_ concentrations, as low as 25 µm, in conjunction with 0.25 µg mL^−1^ of horse radish peroxidase (HRP), elicited a distinct and discernible color shift. The mats were shown to be cytocompatible in vitro with human keratinocytes. In vivo investigations utilizing a non‐chronic wound diabetic (Db/Db) mouse model showed visual changes in mat coloration, transitioning from white to red after a 24‐h incubation interval, corroborating the mats' efficacy in accurately quantifying oxidative stress within these wounds. The H_2_O_2_ sensing mats introduced a straightforward yet impactful approach for dynamically tracking oxidative stress and ROS levels directly at wound sites, heralding a novel dimension in wound monitoring and personalized intervention strategies. However, it is important to highlight that the sensor's efficacy in an in vivo context, particularly within a chronic wound animal model, remains an area that requires further investigation.

#### 3.1.4 Chitosan‐Based Wound Biosensors

Chitosan (CS), derived from chitin, holds tremendous promise in healthcare applications due to its non‐toxicity, biodegradability, electronic conductivity, and mechanical properties.^[^ ^65^, ^66^ ^]^ CS exhibits excellent anti‐microbial properties and can promote cellular growth, migration, and tissue regeneration, making it an attractive choice of material for wound care applications. Due to the unique combination of properties, CS can effectively facilitate all the stages of wound healing, and hence potentially enhance the overall functionality of fabricated wound biosensors.

While the research on CS‐based wound biosensors is currently limited, its potential is remarkable. One group successfully created a composite biosensor using CS for point‐of‐care bacterial infection detection, while another study demonstrated the feasibility of CS‐based biosensors for monitoring diabetic wounds.^[^ ^67^, ^68^ ^]^ The former study introduced a point‐of‐care technology that harnesses lectin‐functionalized chitosan nanoparticles embedded with crystal violet to effectively detect clinically relevant bacterial infections. Characterized by a diameter of <200 nm, these nanoparticles served as the core biosensing nanomaterial. When encountering bacteria, they exhibited distinctive clumping behavior, leading to visible aggregates that stand in contrast to nonbacterial samples. This visible agglutination acted as a definitive indicator of bacterial presence. The technology showcased its prowess by requiring only 100 µL of sample, and it underwent comprehensive testing across a range of bacteria‐spiked environments, including saline, simulated urine, artificial sputum, respiratory swabs, and wound swabs. The in vitro results, obtained from simulated samples spiked with bacteria, demonstrated remarkable sensitivity and specificity, achieving a remarkable detection limit of 10^5^ CFU mL^−1^ (Figure 5D). This innovative point‐of‐care technology significantly advanced the field of infection detection, offering streamlined, easily accessible, and swift diagnostics with substantial potential for practical medical implementation.^[^ ^67^ ^]^ However, investigations into the cytocompatibility of this platform as well as in vivo testing in a chronic wound animal model are warranted.

The other study introduced a biosensor array composed of ultra‐thin Chitosan‐Graphene Oxide (CS‐GO) layers, featuring gold‐based (60 µm) electrodes. The integration of cross‐linked GO played a pivotal role by enhancing the stability of the chitosan substrate within aqueous environments, while concurrently ensuring compatibility with microfabrication processes. The biosensor patch's performance was assessed through label‐free monitoring, involving the immobilization of human dermal fibroblast (HDF) cells on the CS‐GO surface. Cyclic voltammetry (CV) of the HDF cell‐immobilized CS‐GO surface demonstrated consistent peak enhancement during the HDF cell growth period (0–96 h) (Figure 5E). This enhancement exhibited a direct correlation with cell proliferation rates over time, thereby accentuating its potential utility in assessing the dynamic cyto‐physiological status under a spectrum of stimulations, both endogenous and exogenous. Moreover, the sensor patch exhibited the capability of accurately measuring a wide glucose concentration, spanning from 1 to 20 mm, achieving a sensitivity of 0.17 µA mm ^−1^. Collectively, these findings underscored the vast potential of the presented sensor patch in diverse applications, including glucose level detection, monitoring cell health and proliferation rates at wound sites, and aiding in diabetic wound assessment.^[^ ^68^ ^]^ However, it is pertinent to acknowledge the absence of in vivo assessments of the sensor patch in a chronic wound animal model, which could pave the way for further investigations.

#### 3.1.5 Cellulose‐Based Wound Biosensors

Cellulose, the most abundant polymer on earth, can be obtained from plant and bacterial sources.^[^ ^69^ ^]^ Its advantageous properties, such as biodegradability, biocompatibility, and low cytotoxicity, make it highly suitable for a wide range of biomedical applications, including biosensors and wound dressings.^[^ ^70^, ^71^ ^]^ Particularly, cellulose boasts high absorption, swelling rate, exudate retention, versatility, and flexibility, making it advantageous for wound‐related applications. The tunability of physical, chemical, mechanical, and biological properties of cellulose enables its utilization in the fabrication of diverse tailorable biosensors, including wound biosensors.

Several studies have explored cellulose‐based biosensors for wound monitoring. For example, one study discussed a colorimetric approach employing peptide‐conjugated cotton cellulose nanocrystals (CCN) for detecting human neutrophil elastase (HNE), a key biomarker in chronic wounds.^[^ ^72^ ^]^ The paper introduced a colorimetric approach for detecting HNE using peptide‐conjugated CCN. The methodology involved the covalent attachment of a specific HNE tripeptide substrate, n‐Succinyl‐Alanine–Alanine‐Valine‐para‐nitroanilide (Suc‐Ala–Ala‐Val‐pNA), to glycine‐esterified CCN. Notably, the CCN tripeptide conjugates exhibited markedly increased visible HNE activity in comparison to analogous structures on paper substrates. Upon the enzymatic release of para‐nitroaniline (pNA) from the glycine‐CCN conjugate of succinyl‐Ala–Ala‐Val‐pNA, the colorimetric response was amplified by reactive dyes, thereby augmenting the chromogen absorption. The sensitivity of colorimetric HNE detection using CCN tripeptide conjugates was demonstrated at levels consistent with chronic wound fluid (0.05 U mL^−1^ HNE). While this research contributed valuable insights to the evolving field of cellulose‐based colorimetric HNE detection, offering potential applications in wound care and beyond, it is important to note that the system's cytocompatibility and performance within an in vivo chronic wound animal model have yet to be evaluated. Such further investigation would provide crucial insights into the applicability and efficacy of this detection system in real‐world wound healing scenarios.

Another study introduced a peptide‐cellulose biosensor for detecting elevated human neutrophil elastase (HNE) in chronic wounds.^[^ ^73^ ^]^ A comprehensive exploration of a peptide‐cellulose conjugate biosensor, leveraging the exceptional properties of TEMPO‐oxidized nanofibrillated cellulose (tNFC), was reported in the study with the primary aim of discerning elevated levels of human neutrophil elastase (HNE) prevalent in chronic wound scenarios. At the heart of this biosensor design was the strategic attachment of a fluorescent peptide HNE substrate, specifically n‐succinyl‐Ala‐Pro‐Ala‐7‐amino‐4‐methyl‐coumarin, to the TEMPO‐oxidized cellulose surface through the employment of a polyethylene glycol linker. A relatively diminutive crystallite size of tNFC was shown, which distinguished it from its cellulose‐based counterparts, and consequently bestowed upon it a significantly expanded specific surface area as well as crystallite volume ratio. This distinctive structural attribute assumed a pivotal role in enhancing HNE's access to enzyme substrates by mitigating steric hindrances. Further elevating the significance of tNFC, the material's intrinsic porosity took center stage, especially when compared against alternative substrates. This facet underscored tNFC's unique hydrogel‐like nature, amplifying its potential as a substrate for biosensor applications. A particularly intriguing juxtaposition laid in the distinctive attributes of tNFC vis‐à‐vis alternative cellulose‐based materials, ultimately highlighting tNFC's capacity to optimally utilize spatial dimensions and accommodate an augmented assemblage of sensors per crystallite unit volume. The fusion of a modest crystallite volume with an elevated sensor count within the framework of the tNFC peptide‐cellulose conjugate biosensor promised to confer exceptional sensitivity. Consequently, this biosensor not only emerged as a potent contender for the development of tailored point‐of‐care diagnostic tools engineered to discern heightened protease levels, notably Human Neutrophil Elastase (HNE), in the context of chronic wounds but also held promise for broader applications. It is important to acknowledge that further investigations concerning the cytocompatibility of this material, as well as its performance within the complex milieu of an in vivo chronic wound animal model, are yet to be addressed.

Furthermore, researchers have created functional scaffolds by combining cellulose nanocrystals (CNCs) and poly (vinyl alcohol) (PVA) for efficient immobilization of biological probes.^[^ ^74^ ^]^ The study reported the feasibility of transforming CNCs/PVA nanocomposites into robust, water‐insoluble scaffolds with a considerable surface area via a straightforward dip‐coating method, followed by drying and annealing steps. This approach capitalized on the abundant surface hydroxyl groups inherent in both CNCs and PVA, which can be endowed with acrylate functional groups. This augmentation laid the groundwork for the subsequent incorporation of a substantial concentration of fluorescent sensor motifs through gentle thiol–ene addition reactions. The developed sensor films achieved rapid and prominent changes in fluorescence emission intensity with shifts in pH, offering an almost instantaneous response. This strategy was further utilized to encompass the detection of protease activity. By introducing a Förster‐type resonance energy transfer chromophore pair via a labile peptide sequence into the scaffold, the cleavage of the protein linker in the presence of pertinent enzymes was harnessed. This cleavage event effectively triggered the separation of the chromophores, inducing a ‘turn‐on’ effect that elevated the initially quenched fluorescence to heightened intensity levels. Employing a conventional benchtop spectrometer to quantify the elevation in fluorescence intensity, the presence of trypsin was effectively identified at concentrations typically indicative of abnormal proteolytic activity, a common feature observed in wound fluids. The integration of CNCs and PVA within these multifunctional scaffolds not only underscored their versatile potential in fluorescence‐based sensing applications but also laid the groundwork for future exploration. However, it is imperative to acknowledge that key aspects, such as the platform's cytocompatibility and its performance within the intricate context of an in vivo chronic wound animal model, remain avenues for further investigation and development.

### 3.2 Synthetic Polymers

#### 3.2.1 Polyurethane‐Based Wound Biosensors

Polyurethane (PU), known for its biocompatibility and biodegradability, finds extensive use in medical fields, including wound dressings and bio‐adhesives.^[^ ^75^, ^76^ ^]^ PU has excellent water absorption and retention capacity, besides being gas permeable, flexible, and elastic. Its versatility enables the delivery of growth factors and antibiotics, promoting wound healing and controlling infections by forming a protective barrier against bacteria.^[^ ^77^ ^]^ PU also offers a high degree of tunability of physical, chemical, and mechanical properties and has been utilized for fabricating biosensors for wound monitoring.

State‐of‐the‐art studies have demonstrated the potential of PU‐polydopamine (PDA) nanofiber composites in biosensor applications, particularly in smart wound dressings for point‐of‐care use.^[^ ^78^, ^79^ ^]^ In one of the studies,^[^ ^79^ ^]^ introduced a straightforward approach through electrospinning to fabricate receptor‐free polyurethane (PU)‐integrated polydiacetylene (PDA) nanofiber biosensors. These nanofibers exhibited rapid color transitions from blue to red, serving as a sensitive means to detect the presence of *E. coli*. This biosensor utilized electrospinning to create nanofibers that bypass the requirement for intricate receptors in *E. coli* detection based on colorimetric responses. The process of electrospinning triggered the autonomous assembly of diacetylene monomers, significantly enhancing the interaction between PDA macromolecules and *E. coli*. This heightened interaction translated into remarkable sensitivity for detecting bacterial presence. Of particular note, the PU‐PDA nanofiber system demonstrated exceptional sensitivity, enabling swift and discernible color transitions that can be easily perceived by the naked eye when *E. coli* is present. This multifaceted biosensor design held promise for diverse applications and opened avenues for future explorations into extracellular polymeric substances (EPS)‐based analyses, marking a significant contribution to the field of biosensing and detection technologies. However, key aspects, such as the platform's cytocompatibility and its performance in an in vivo chronic wound animal model, are yet to be studied.

**Figure 6:**
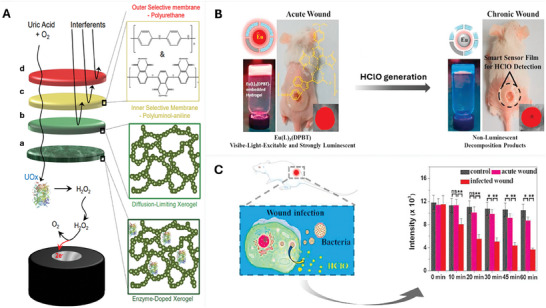
Synthetic polymer‐based systems for wound biosensing. Polyurethane‐based biosensor for uric acid detection using a layer‐by‐layer approach. A) Schematic illustration of the four‐layer structure of the biosensor on a platinum electrode. The layers are a) uricase‐doped hydroxymethyltriethoxysilane (HMTES), b) undoped HMTES xerogel for enzyme immobilization, c) polylysine‐anthraquinone (PL‐A) electropolymer for electrocatalytic activity, and d) polyurethane (PU) membrane for selective permeability. Reproduced with permission.^[^ ^80^ ^]^ Copyright 2016, Elsevier. PEG hydrogel‐embedded smart sensor films for wound infection detection. B) The immobilization of the probe Eu(L)_3_(DPBT) in PEG hydrogel allows for a smart sensor film whose luminescence changes with the concentration of hypochlorous acid (HClO). The film detects HClO in wound exudates and differentiates between infected and acute wounds. Reproduced with permission.^[^ ^87^ ^]^ Copyright 2020, Elsevier. C) A schematic illustration of HCIO production by bacteria in infected wounds and quantified luminescence intensities from the affected regions. Data presented as mean ± standard deviation for three replicates. (*p < 0.05, **p < 0.01, ns: non‐significant vs control group). Reproduced with permission.^[^ ^87^ ^]^ Copyright 2020, Elsevier.

Additionally, a pioneering biosensor for uric acid (UA) has been developed using a layer‐by‐layer (LbL) approach, designing an electrode with specific polymeric and xerogel materials.^[^ ^80^ ^]^ Specifically, the study introduced an innovative fabrication method for developing a first‐generation amperometric biosensor tailored to detect UA through an LbL approach. The research entailed a comprehensive analysis and refinement of each distinct layer, encompassing an outer polyurethane (PU) membrane engineered for selectivity, an inner electropolymer distinguished by its selective properties, and a dual‐layer xerogel structure. This systematic exploration not only elucidated the influence of PU hydrophobicity on UA permeability but also imparted crucial insights into the contribution of each layer to the overall biosensor performance. The culmination of this investigation yielded a highly optimized combination that seamlessly incorporated hydroxyl‐methyl triethoxy silane (HMTES) xerogels, a polyluminol‐aniline electropolymer, and 100% hydrophobic polyurethane, resulting in significantly heightened UA sensing capabilities (**Figure** **6**A). These advancements were manifested in heightened sensitivity, quantified at 0.8 nA µm ^−1^, alongside a linear response range spanning the physiologically relevant UA concentration spectrum (100–700 µm). The biosensor exhibited rapid response times of ≈10 s, low thresholds of detection (<10 µm), and an exceptional level of selectivity against common interferents. Through meticulous tailoring of the LbL architecture and strategic utilization of interactions among thoughtfully selected materials, this research laid a robust foundation for the advancement of cutting‐edge biosensors. Nonetheless, it is worth noting that there remains an avenue for further research to explore the cytocompatibility of this platform and assess its performance within an in vivo chronic wound animal model. Addressing these aspects will serve to enhance the comprehensiveness of this study and underscore the broader potential of this biosensing approach.

#### 3.2.2 Polyvinyl Alcohol‐Based Wound Biosensors

Polyvinyl alcohol (PVA) hydrogels have gained significant interest in biomedical applications, including temporary skin covers and burn dressings.^[^ ^81^ ^]^ Their appeal lies in their non‐toxicity, excellent biocompatibility, elastic nature, and high water absorption capacity.^[^ ^20^ ^]^ Other desirable properties, such as high water uptake and retention, excellent processability, chemical resistance, and high mechanical strength make PVA an appropriate material for wound applications.

In the context of wound biosensors, PVA has often been used in conjunction with other polymers for the detection of wound biomarkers. For example, researchers developed an innovative electrochemical approach for wound monitoring using uricase as an enzyme to detect uric acid (UA).^[^ ^82^ ^]^ The study presented an electrochemical methodology that employs uricase (UOx) as an enzyme for the specific purpose of detecting UA, with a specific focus on its application in wound monitoring. To achieve this, UOx was encapsulated within a cationic polymer matrix, specifically poly (vinyl alcohol) N‐methyl‐4(4’‐formylstyryl) pyridinium methosulfate acetal (PVA‐SbQ). In order to facilitate efficient electron transfer, a redox electron shuttle, ferrocene carboxylic acid (FCA), was introduced. The entrapped UOx showcased significantly enhanced responsiveness in detecting UA, distinctly surpassing the performance of physisorbed UOx. The sensor's response demonstrated linearity across physiologically relevant concentration ranges, particularly within the range of 12–100 µm. Furthermore, the stability of the entrapped UOx biosensor was systematically evaluated over a span of 48 h, with a remarkable retention of 90% activity sustained for an extended duration of up to 5 days. The applicability of this entrapped biosensor was demonstrated through the successful measurement of UA levels in biofluids derived from sweat and wound samples. Notably, the sensor exhibited a robust recovery rate spanning ≈102–107%. The culmination of this pioneering research held significant potential, offering a direct and reliable avenue for measuring uric acid—a pivotal biomarker for evaluating wound chronicity. The amalgamation of enzymatic immobilization, efficient electron transfer, and the capacity for continuous monitoring positioned this biosensor as a transformative tool with versatile applications in the realm of clinical diagnostics, particularly within the domain of wound management. While this work presented a substantial leap forward, it is essential to address further aspects such as the cytocompatibility of the platform and its performance in an in vivo chronic wound animal model.

In another study, to create a smart wound dressing with antibacterial and biochromic properties, an anthocyanin probe was incorporated into a composite of carboxymethyl cellulose and PVA, along with potassium aluminum sulfate mordant.^[^ ^19^ ^]^ The study reported an aerogel‐like wound dressing that possesses the unique capability to monitor wound healing progression. This innovative bioassay proficiently detected pH fluctuations within simulated wound solutions, enabling the identification of colorimetric changes. The integration of an anthocyanin probe, as a water‐soluble direct dye, into a composite of carboxymethyl cellulose/polyvinyl alcohol was achieved through the strategic introduction of a potassium aluminum sulfate mordant. The adsorption of the anthocyanin indicator into the matrix of carboxymethyl cellulose and polyvinyl alcohol (CMC/PVA) intricately facilitated the sensory process. This intelligent dressing emerged as a multifaceted biosensor, orchestrating real‐time monitoring of wound healing progression. The introduction of a wound‐mimicking fluid featuring a reduced pH value instigated a hypsochromic shift, causing the absorption spectrum to transition from 592 to 446 nm. This halochromic reaction of the anthocyanin yielded discernible colorimetric alterations, transitioning from an initial purple hue to a striking pink tint. Additional investigations were conducted to assess the cytotoxicity and antibacterial attributes intrinsic to the evolved aerogel‐like dressing. This research represented an advancement in the domain of wound care technology, orchestrating the harmonious integration of wound healing monitoring and antibacterial efficacy within a single entity. This achievement heralded a transformative milestone in the evolution of wound dressing design, ushering in a chromogenic sensor that not only enhanced wound monitoring capabilities but also underscored the immense potential inherent in harnessing nature's reservoirs for groundbreaking medical diagnostics. While this research heralded a significant stride, its application to a chronic wound animal model for in vivo evaluation remains an avenue that warrants future exploration and investigation.

#### 3.2.3 Polyethylene Glycol‐Based Wound Biosensors

Polyethylene glycol (PEG) is a hydrophilic and biocompatible polymer known for its neutral behavior in aqueous solutions, making it ideal for various applications, including drug delivery and tissue engineering.^[^ ^83^ ^]^ PEG hydrogels have gained popularity as a core material for biosensors and tissue engineering due to their ability to reduce nonspecific binding of proteins and cells.^[^ ^84^ ^]^ PEG also offers easily tunable mechanical properties and immunomodulatory attributes, besides being resistant to systemic degradation and clearance.

Recently, a novel biosensor was introduced, combining a polyethylene glycol (PEG) hydrogel with a fluorescence resonance energy transfer (FRET)‐peptide to detect protease activity.^[^ ^85^ ^]^ The study reported the employment of photolithography to immobilize a FRET peptide onto a micropatterned hydrogel substrate made of polyethylene diacrylate (PEGDA). The prime objective was to target the prominent enzymes MMP‐2 and ‐9, crucial for gauging wound severity. By immobilizing the FRET‐peptide sequence Dabcyl‐Gly‐Pro‐Leu‐Gly‐Met‐Trp‐Ser‐Arg‐Lys (FITC)‐Cys, susceptible to cleavage between Gly and Met in the presence of MMP‐2 or MMP‐9, the ensuing amplification in hydrogel fluorescence emerged as a quantifiable measure of protease concentration. This hydrogel‐based biosensor exhibited substantial potential for detecting vital proteases within chronic wounds. However, avenues remain for further exploration, encompassing the cytocompatibility of this platform and its efficacy in a chronic wound animal model in vivo.

In another study, the PEG hydrogel was employed to immobilize a probe for the creation of smart sensor films capable of swiftly responding to hypochlorous acid (HClO).^[^ ^86^ ^]^ The study introduced a novel luminescence probe, Eu(L)3(DPBT), based on Eu^3+^ complexes, designed and synthesized to enable time‐gated luminescence (TGL) detection of HClO both in vitro and in vivo (Figure 6B). This probe exhibited rapid, exceptional selectivity, and high sensitivity in TGL responsiveness to HClO while maintaining low cytotoxicity. Moreover, through the incorporation of the Eu(L)3(DPBT) probe into a PEG hydrogel matrix, innovative sensor films were successfully engineered. These films can be applied to the skin's surface, enabling real‐time monitoring of HClO generation in non‐chronic wounds in mice, thereby facilitating the distinction between infected and acute wounds (Figure 6C). This pioneering approach held promise as a diagnostic and therapeutic tool for managing wound‐related conditions. However, it is crucial to acknowledge that the quantification of intracellular HClO presents challenges due to the complex interplay of sample conditions, local probe concentrations, and excitation fluctuations. Additionally, it's noteworthy that the assessment of this biosensor in a chronic wound animal model remains an avenue for future exploration.

#### 3.2.4 Polycaprolactone‐Based Wound Biosensors

Polycaprolactone (PCL), a synthetic aliphatic polyester, is known for its biodegradability, biocompatibility, and versatile physical properties, including high elasticity, low melting point, and ductility.^[^ ^87^ ^]^ PCL offers excellent tunability and processability, high mechanical strength, and can be easily incorporated as a base material for biosensor fabrication.

In the domain of biosensing applications, one study reported surface‐functionalized electrospun PCL nanofibers, possessing a large surface‐area‐to‐volume ratio allowing for enhanced surface modification, with carbon quantum dots (CQDs) to create a strong fluorescence signal.^[^ ^88^ ^]^ The study introduced an electrospun biosensor, engineered to harness aptamers and the distinctive fluorescence of CQDs, for rapid, cost‐effective, portable, and sensitive detection of *S. aureus* bacteria within wound environments. Synthesized from orto‐phenylenediamine (OPD), the CQDs exhibited distinctive yellow emission fluorescence. The integration of luminescent CQDs into polymeric nanofibers (NFs) was achieved via cross‐linking. This accomplishment was realized through the successful electrospinning of fluorescent PCL‐CQD NFs, revealing the incorporation of CQDs within the NF matrix through interactions with the polymer chains. The engineered NF aptasensor, characterized by its selectivity, reproducibility, stability, and cytocompatibility with fibroblast L929 cells, stood as a solution enabling direct identification of bacteria within infected wounds. Notably, the application of modified NFs to non‐chronic skin wounds in mice showcased an amplified fluorescence intensity under UV light after 2 h, underlining the successful deployment of the aptasensor. Of significance, the platform offered a linear sensing range spanning an impressive 10–10^8^ CFU mL^−1^, with a remarkable detection limit of 10 CFU mL^−1^. Furthermore, this study hinted at the versatility of the proposed platform, indicating its adaptability to diverse microbial or viral entities through appropriate bio‐receptor incorporation. Nevertheless, it's essential to underscore the need for additional exploration to validate the aptasensor's performance across various wound scenarios and its potential clinical implications.

All in all, the integration of natural and synthetic polymers in wound biosensing and management has led to significant advancements in wound care. These innovative materials and biosensors offer great promise in improving wound monitoring, infection detection, and wound healing outcomes. As research continues to evolve, the combination of polymer‐based biosensors with smart technologies holds the potential to revolutionize wound care practices and enhance the quality of life for patients.

## 4 Animals and Biosensors

One of the key requirements for successful clinical translation of newly developed wound biosensors is comprehensive validation of the biosensors in an in vivo setting. Although there are several animal studies testing biosensors in non‐chronic and/or non‐diabetic wound models, there is a distinct shortage of pre‐clinical animal testing of recently developed polymeric wound biosensors in diabetic chronic wound models. Our review highlights the urgent need for biosensor testing and validation in chronic wound animal models since diabetic chronic wounds are a growing healthcare concern. Nonetheless, a summary of the existing literature highlighting the different animal models for wound biosensor testing has been discussed in the following section.

### 4.1 Rodent Models

Small animal models, such as mice and rats, have been commonly used in biosensor studies for wound healing. These studies often involve creating standardized wounds on the animals and then monitoring the healing process using biosensors. Biosensors may be implanted or attached to the wound site to measure parameters such as pH, oxygen levels, temperature, or specific biomarkers. The VeCare dressing described previously under biomarker sensing combined biosensors for inflammatory mediators like tumor necrosis factor α (TNF‐α), interleukin‐6, interleukin‐8, growth factor TGF‐β, detection of *S.aureus* and temperature and pressure in venous ulcers by analysis of the wound fluid. This dressing was tested on full‐thickness excisional wounds in mice. Ten to twelve‐week‐old, male Institute of Cancer Research outbred mice were used for the study. The wounds were monitored along with control wounds (No VeCare) for 5 days and the biomarkers showed appropriate variation (pH became more acidic compared to initial alkalinity at Day 0) once re‐epithelialization began.^[^ ^30^ ^]^ It should be noted that this line is not in live production.^[^ ^20^ ^]^ designed a smart dressing that measures metabolic and inflammatory biomarkers (glucose, uric acid, lactic acid) via electrochemical signals from wound fluid. The amperometric and potentiometric electrodes for measurement and drug delivery/treatment respectively are fabricated on a poly[styrene‐b‐(ethylene‐co‐butylene)‐b‐styrene] (SEBS) substrate making it flexible. Data collected by the biosensors was transmitted through wireless communication. The efficacy of the patch for drug delivery was tested in vivo on diabetic murine wound models. A thrombin‐derived c‐terminal peptide‐25 (TCP‐25), which has anti‐microbial and anti‐inflammatory properties, was loaded into the chitosan hydrogel on the substrate which released the drug on the application of positive voltage. Diabetic mouse models were used to test the efficacy of biomarker measurement before and after bacterial infection. The antimicrobial activity of TCR‐25 was evaluated against methicillin‐resistant *S. aureus*, *P. aeruginosa*, *E. coli*, and *Staphylococcus epidermidis* as these species are most abundant in diabetic foot ulcers. The wearable patch noted an increase in uric acid, temperature, pH, and lactate and a decrease in glucose levels following infection while the reverse was noted following a subsequent treatment with electrical stimulation. Zucker diabetic fatty rats were used to test the efficacy of drug delivery. Best results were obtained in a 14‐day monitoring period by administering TCP‐25 in addition to electrical stimulation which resulted in most closed wounds in rats.^[^ ^89^ ^]^

### 4.2 Porcine Models

Pigs are frequently used as a preclinical model due to their anatomical and physiological similarities to human skin. Biosensors have been employed in porcine models to monitor wound healing parameters, including oxygenation, pH levels, bacterial presence, and inflammatory markers. These studies provide valuable insights into the efficacy and performance of biosensors in a larger animal model.^[^ ^21^ ^]^ fabricated pH‐sensitive hydrogel fibers. Hydrogel fibers created by microfluidic spinning were loaded with mesoporous microparticle particles that had pH‐sensitive dye incorporated into them. This hydrogel was then applied to adhesive medical tape and tested on pig skin. The varying wound in the pH was simulated by spraying the explanted pig skin with agarose gel at different pH levels. A micrograph read by a custom MATLAB program designed to decode the color change in the pH‐sensitive hydrogel showed distinct differences in color at pH 6.2, 7.2, and 8.2.^[^ ^21^ ^]^ However, the use of pig skin in this study is *ex vivo* and leaves room for testing in vivo.^[^ ^90^ ^]^ utilized an oxygen‐sensing paint to interpret skin inflammation levels in a porcine inflammation model. The paint was formulated by dissolving Pd‐meso‐tetra‐(4‐carboxyphenyl) porphyrin dendrimer in p‐toluenesulfonic acid monohydrate in refluxing ethanol. This oxygen‐sensing paint was mixed with liquid bandage and applied to the wound area followed by a transparent bandage like Tegaderm. Wound areas or inflamed injection sites were created by injecting mycobacterium tuberculosis intradermally into MHC‐defined miniature swine. Inflamed tissue consumes a significantly higher amount of oxygen compared to non‐inflamed skin and the results of the study from the oxygen‐sensing painted bandage reflected as much.^[^ ^90^ ^]^ In another study done by^[^ ^29^ ^]^ oxygenation in the wound was measured in both murine and porcine wound models. The sensor was fashioned as dual‐layer films of oxygen‐sensing boron nanoparticles (BNPs) layered on chitosan‐polycaprolactone on top of a calcium alginate layer for flexibility in maximizing wound bed contact. When BNPs in the sensor detect oxygen, they emit phosphorescence (P). Additionally, when UV light is incident on BNPs they emit an oxygen‐independent fluorescence (F). The F/P ratio is then evaluated to gauge the oxygenation of the wound. Female C57BL6 mice were operated on to create a full‐thickness dermal wound and one female domestic Yorkshire swine was operated on to create a full‐thickness excisional wound. Both were treated with the BNP‐based biosensor. The wounds were monitored photographically by brightfield and UV imaging. The study concluded that the application of the thin film of BNPs in both in vitro studies helped in the easy mapping of oxygen levels throughout the wound bed as it maintains contact with the entire wound irrespective of position on the body shape. However, the authors do mention that due to the small sample size, the in vitro studies should be repeated with a larger sample size and more elaborate time‐courses to observe healing.^[^ ^29^ ^]^ As porcine skin is physiologically closer to human skin, the in vivo efficacy of biosensors with porcine models can provide crucial data on the suitability of the innovation.

### 4.3 Non‐Human Primates

In certain cases, non‐human primates, have been used in biosensor studies for wound healing. Primates provide a closer resemblance to human wound healing processes and allow for the evaluation of biosensors in the context of host recognition and immune‐mediated foreign body response. For example, alginate specifically may trigger a multi‐immune population foreign body response. The response is due to the presence of endotoxins as a by‐product of the manufacturing process as well as to clinical‐grade alginate with no detectable levels of endotoxins. However, we couldn't find any studies specific to wound healing monitored with biosensors in non‐human primates.

These animal studies involving biosensors for wound healing aim to assess the performance, accuracy, and reliability of the sensors in real‐time wound monitoring. They provide valuable data on the feasibility and potential benefits of using biosensors for clinical applications in humans. It's important to note that each study may focus on different aspects and specific parameters relevant to wound healing, depending on the goals and objectives of the research.

## 5 Clinical Translation of Wound Biosensors

Current methods for assessing wound healing predominantly rely on visual inspection of the wound area, evaluating parameters such as microbial infection, re‐epithelialization, and granulation tissue formation. These assessments are often subjective and heavily dependent on the clinician's expertise. While quantitative wound assessment modalities do exist, they are typically time‐consuming and costly due to the requirement for laboratory investigations. In this context, the development of smart, wearable, advanced, and real‐time wound monitoring biosensors is crucial for the optimal care and management of wounds, particularly for chronic, non‐healing wounds. These innovative biosensors have the potential to revolutionize wound care by providing continuous, objective data, thereby facilitating timely and precise interventions. However, the clinical translation of these advanced wound biosensors faces significant challenges. A comprehensive safety and efficacy evaluation is essential for their transition from the bench to the bedside. The process is often hindered by time and cost constraints associated with the thorough testing and validation required to obtain regulatory clearance. To overcome these challenges, collaborative efforts among researchers, clinicians, industry stakeholders, and regulatory authorities are imperative. Such collaboration should focus on the development of innovative wound biosensors, detailed characterization, and rigorous testing and validation across various models, including in silico, in vitro, and in vivo systems. Furthermore, the path to clinical adoption necessitates the development of commercially viable products that can be seamlessly integrated into routine clinical practice.^[^ ^91^ ^]^ Addressing these challenges and fostering such collaborations will be instrumental in advancing the field of wound care, ultimately improving patient outcomes through the widespread adoption of next‐generation wound biosensors.

## 6 Conclusion

Ongoing research aims to enhance biosensor capabilities in wound monitoring. This includes the development of miniaturized and wearable biosensors, the incorporation of multiple sensing modalities into a single device, and the integration of biosensors with advanced data analytics and artificial intelligence for real‐time wound assessment and personalized treatment recommendations. Several polymers have been used in biosensors for wound healing applications. The trend in the development of biosensors seems to have evolved into multi‐component biosensors and now moving toward smart dressings as well as the implementation of machine learning algorithms in identifying the stage of wound to customize the dressing for patients. This ability to personalize the dressings will greatly benefit patients suffering from chronic wounds. The most used polymers in biosensors designed for monitoring wound healing, include PEG, PU, PLGA, and PVA and are chosen based on various factors such as biocompatibility, mechanical properties, and the specific requirements of the biosensor design and functionality. Overall, the history of biosensors in wound healing applications has evolved from early enzyme‐based sensors to more sophisticated and diverse sensing modalities. Biosensors have become valuable tools in wound management, enabling clinicians to monitor wound parameters, detect complications, and guide treatment decisions for optimal wound healing outcomes.

## Conflict of Interest

The authors declare no conflict of interest.
